# Endogenous Methicillin-Resistant Staphylococcus aureus (MRSA) Endophthalmitis Presenting as an Anterior Chamber Abscess With Necrotizing Scleritis

**DOI:** 10.7759/cureus.108558

**Published:** 2026-05-09

**Authors:** Zach P Dawson, Alec Curtis, Stephen Cross, Adam Pearlman

**Affiliations:** 1 Ophthalmology, Kansas City University of Medicine and Biosciences, Kansas City, USA; 2 Ophthalmology, University of South Carolina/Prisma Health, Columbia, USA

**Keywords:** anterior chamber, endophthalmitis, hypopyon, necrotizing scleritis, staphylococcus aureus

## Abstract

To report a rare presentation of endogenous methicillin-resistant *Staphylococcus aureus* (MRSA) endophthalmitis with necrotizing scleritis manifesting as an anterior chamber (AC) abscess, and to highlight the diagnostic value of repeating AC sampling when clinical suspicion for infection remains high.

A 29-year-old man developed a progressive AC mass and decreased vision one week after incision and drainage of an MRSA thigh abscess. Ocular symptoms progressed for two months before ophthalmic evaluation revealed a large AC lesion with hypopyon and early scleral thinning. An initial AC tap was negative; corticosteroids were associated with clinical worsening. A repeat AC tap demonstrated Gram-positive *cocci*, and cultures confirmed MRSA. Treatment with intravitreal vancomycin and ceftazidime, systemic antibiotics, and tapered corticosteroids led to improvement in visual acuity from light perception to 20/30 3.5 months after the initial eye exam.

Endogenous MRSA endophthalmitis is uncommon and typically posterior; presentation as an AC abscess is rare. When clinical findings strongly suggest infection, repeat diagnostic sampling should be performed despite an initial negative result. Early targeted intravitreal antibiotics and coordinated subspecialty care can preserve vision.

## Introduction

Endogenous endophthalmitis (EE) is a rare, vision-threatening intraocular infection seeded hematogenously from a distant focus; MRSA is a particularly aggressive bacterial cause [1]. EE arises from bacteria entering a previously sterile site from a distant source of infection. EE more often involves the posterior segment and demonstrates a right-eye predilection attributed to vascular anatomy due to more proximal and direct blood flow from the right carotid artery [2]. Presentation as a predominantly anterior process with an AC abscess is unusual. Diagnosis can be difficult as the clinical course may begin with nonspecific signs and symptoms. 

Necrotizing scleritis is another destructive ocular condition, most commonly autoimmune but occasionally infectious [3]. Infectious necrotizing scleritis carries a high risk of perforation if not promptly treated [4].

We report a case of MRSA EE with necrotizing scleritis that initially appeared similar to granulomatous anterior uveitis, even remaining culture-negative on the first AC tap, and was only confirmed as EE after repeat AC paracentesis. The case highlights the limitations of a single negative aqueous result and the importance of diagnostic persistence based on clinical suspicion.

## Case presentation

A 29-year-old man with obesity and prediabetes presented with a painful right thigh abscess requiring incision and drainage; wound culture grew MRSA. He was treated with IV ceftriaxone followed by 10 days of trimethoprim/sulfamethoxazole and cephalexin.

Over subsequent weeks, he re-presented multiple times for persistent leg pain, later diagnosed as superficial thrombophlebitis and treated with apixaban. During one visit, he reported right-eye pain, but no ophthalmic examination was performed.

**Figure 1:**
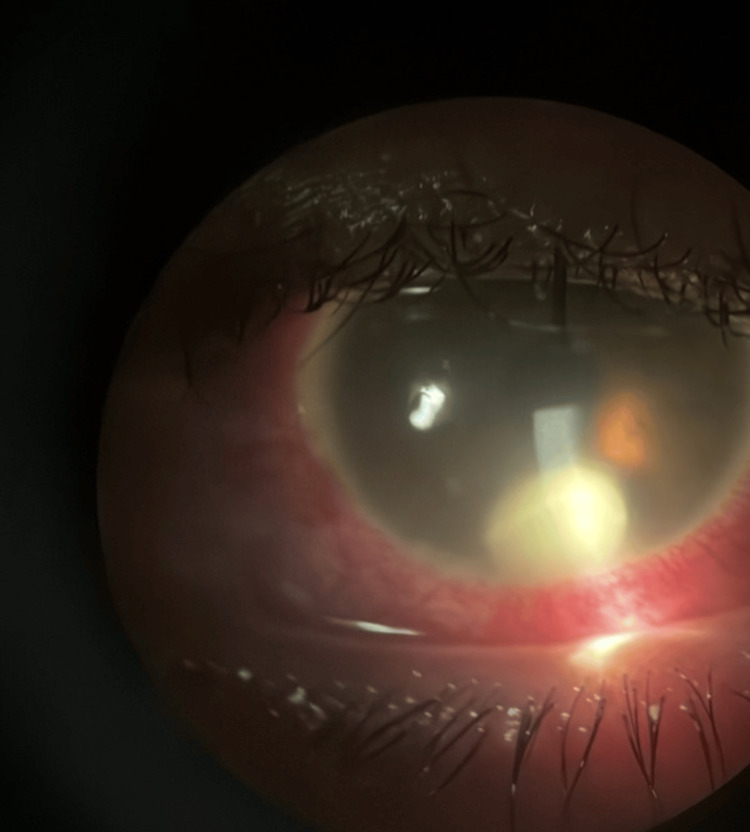
Slit-lamp photos at initial ophthalmic presentation showing conjunctival injection, corneal edema, and large anterior chamber mass.

**Figure 2:**
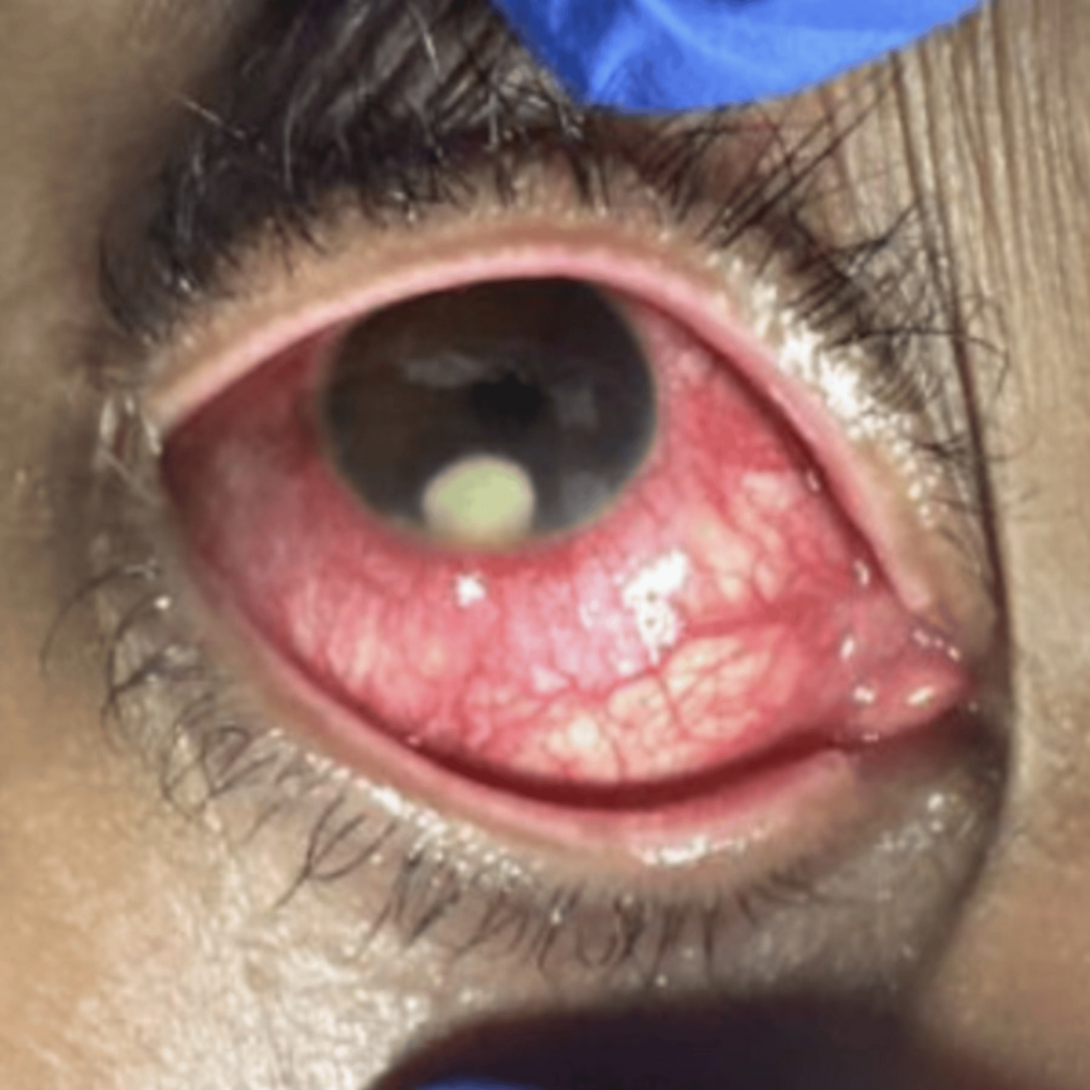
A photo taken via phone camera was sent to ophthalmology by the emergency department.

Two and a half months after abscess drainage, he presented with worsening right-eye pain and decreased vision. Examination showed VA 20/50-1 OD, conjunctival injection, corneal edema, and a large AC mass (Figures 1, 2). Laboratory studies (Table 1) revealed markedly elevated erythrocyte sedimentation rate (ESR) (>130 mm/hr) and C-reactive protein (CRP) (149 mg/L); infectious serologies were negative. He was started on topical moxifloxacin, cyclopentolate, and artificial tears.

**Table 1:** Lab findings. CRP: C-reactive protein, BUN: Blood urea nitrogen, ESR: Erythrocyte sedimentation rate

	Reference Range	Result
CRP Inflammatory	<=0.5 mg/L	149
Sodium	136-145 mmol/L	140
Potassium	3.5-5.1 mmol/L	3.5
Chloride	98-107 mmol/L	103
CO2	22-29mmol/L	26
BUN	6-20 mg/dL	11
Calcium	8.6-10.0 mg/dL	9.2
Creatinine, Serum	0.67-1.17 mg/dL	1.13
Glucose	70-99 mg/dL	118
Anion Gap	6-16 mmol/L	11
eGFR	>59 mL/min/1.73 m2	90
ESR	0-15 mm/hr	>130

**Figure 3:**
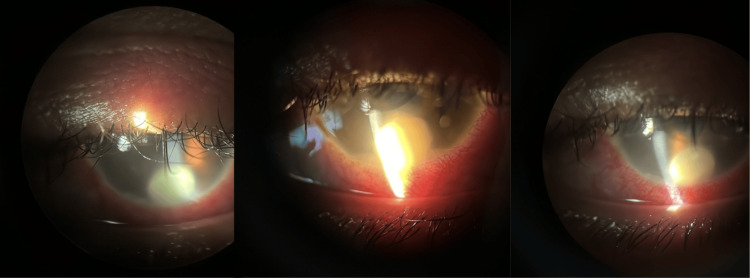
Slit lamp photos on day two in the clinic were notable for hypopyon.

Next day: VA declined to 20/150; IOP rose to 41 mmHg. The slit lamp exam was significant for increasing the size of the hypopyon (Figure 3). Prednisolone acetate 1% was initiated for presumed inflammation, along with IOP-lowering agents.

**Figure 4:**
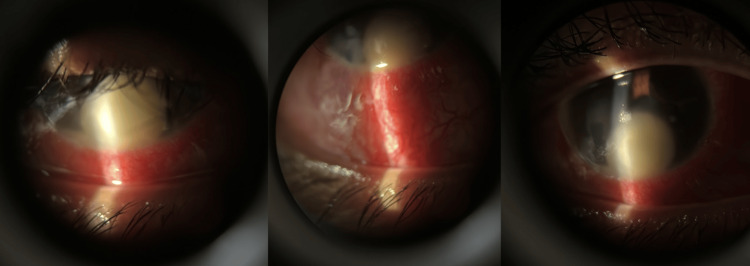
Slit lamp photos on day four showing increased infiltrate size and a new nodule on the inferior sclera.

Three days later: pain worsened, VA dropped to light perception, the AC was filled with fibrin and hypopyon, and nodular scleral thickening with early thinning was noted (Figure 4). The B-scan showed no posterior involvement. Given hypopyon and worsening intraocular inflammation, clinical suspicion remained elevated for an acute infectious process. Oral prednisone 60 mg was started for presumed necrotizing scleritis.

A retina/uveitis specialist performed an AC tap (culture/PCR) and started oral levofloxacin while awaiting results. Prednisone was increased to 80 mg; indomethacin 50 mg twice per day was added. An MRI of the orbits/brain was obtained the next day and was unremarkable.

**Figure 5:**
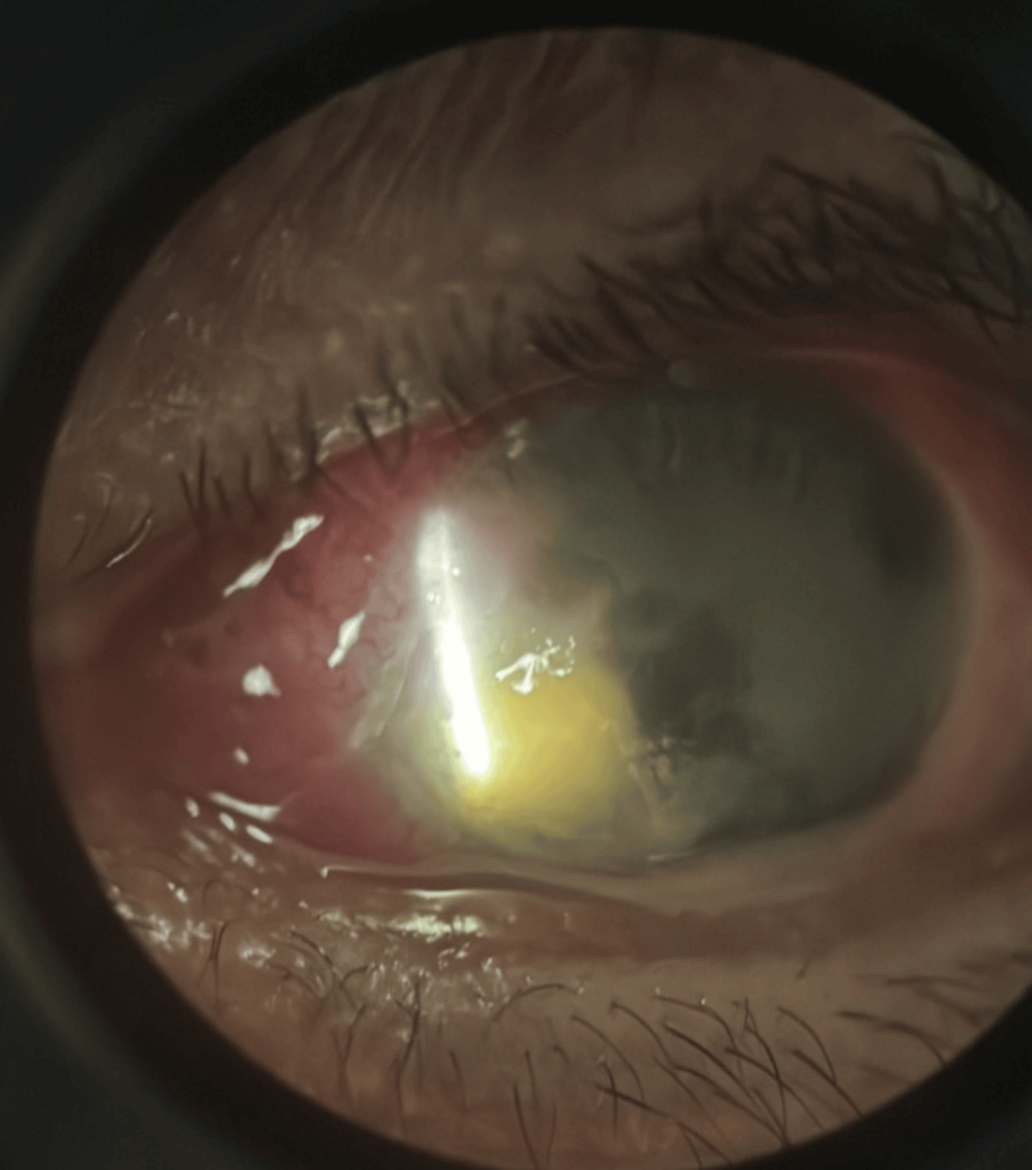
Slit lamp photo on day eight showing displacement of infiltrate after AC tap #1 and slight improvement in scleritis.

**Figure 6:**
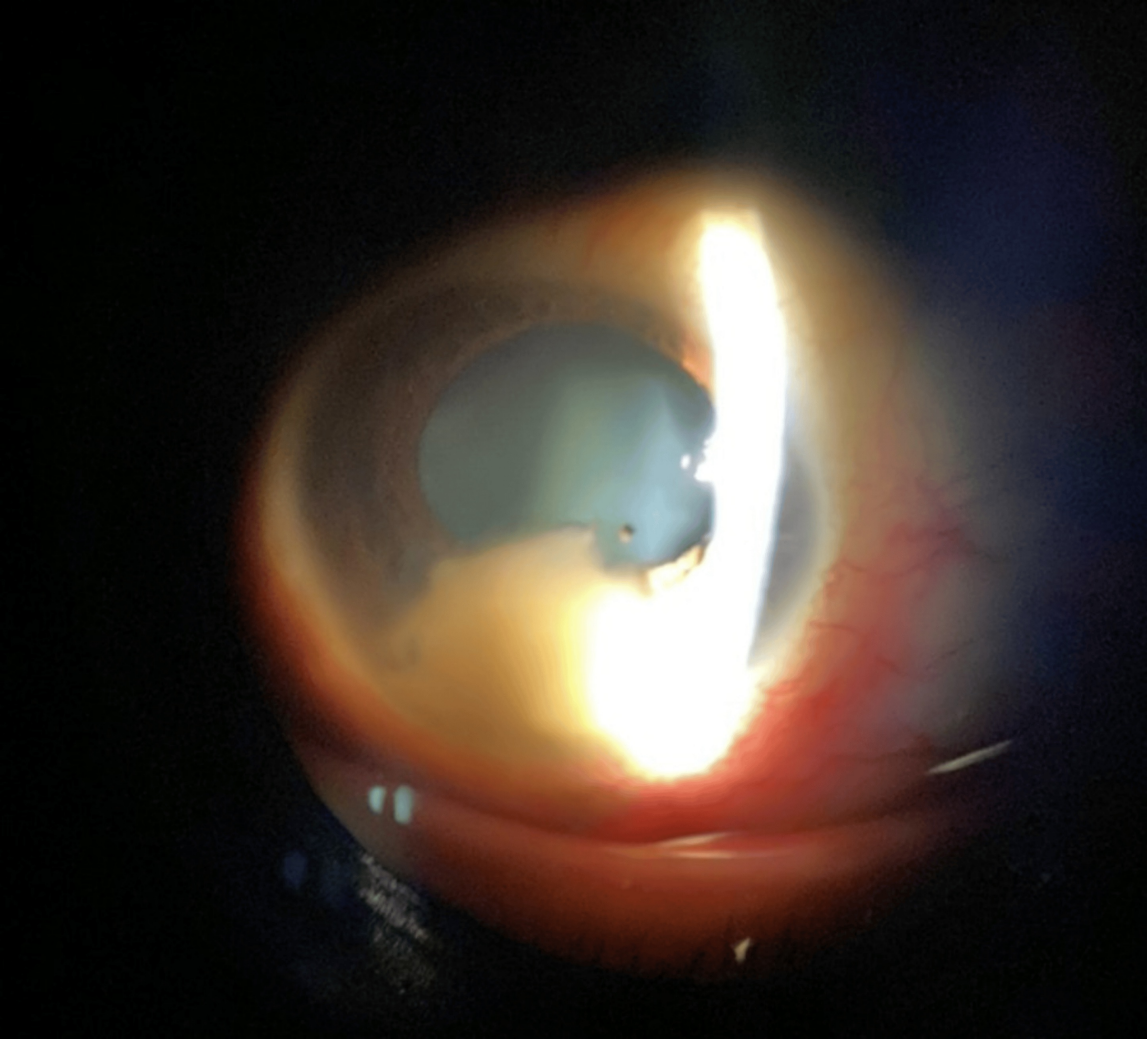
Slit lamp photo on day nine showing significant improvement in scleritis and further disruption of infiltrate immediately after the second AC tap. AC: Anterior chamber

**Figure 7:**
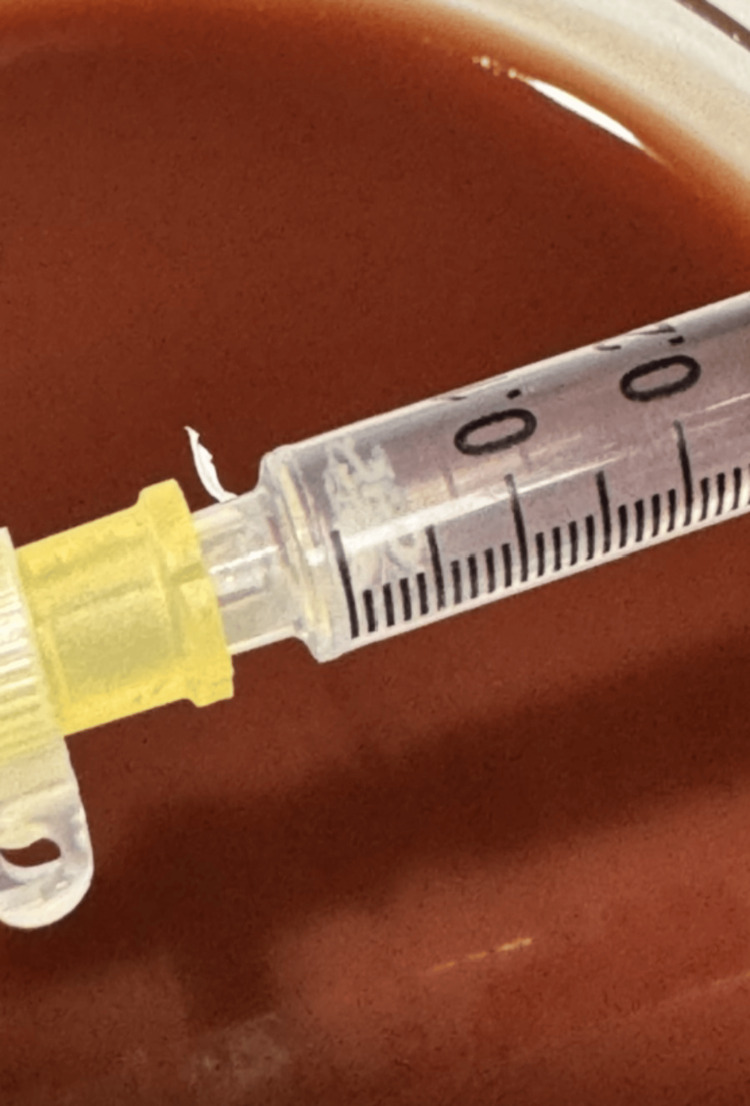
Shows the material from the AC tap #2. AC: Anterior chamber

**Figure 8:**
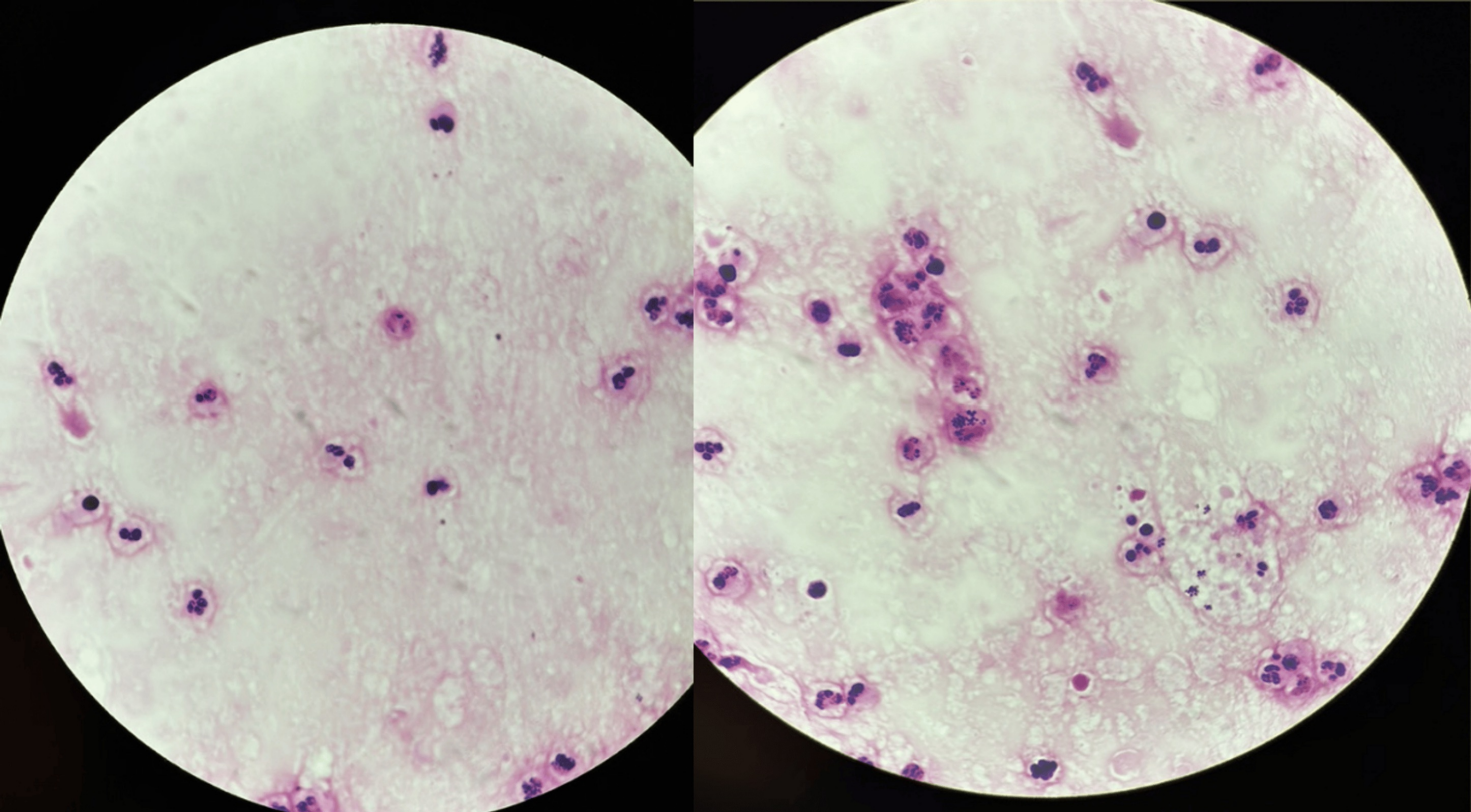
Photos taken at the microscope of a gram stain showing gram-positive cocci; this is also showing PNMs. PNM: Polymorphonuclear leukocytes

Day eight (cornea specialist visit): The first AC culture/PCR returned negative. VA was counting fingers, IOP 36→21 mmHg with drops. The exam showed reduced injection and a shrinking scleral nodule but a more yellow, disrupted AC mass with hypopyon and a new 1.5 mm × 1.5 mm corneal epithelial defect (Figure 5). Given high suspicion for infection, steroids were tapered, and a repeat AC tap was performed (Figures 6, 7). Gram stain showed Gram-positive cocci; culture later confirmed MRSA (Figure 8). The next day, he received intravitreal vancomycin and ceftazidime. Oral prednisone was reduced and tapered over approximately 1.5 weeks. 

**Figure 9:**
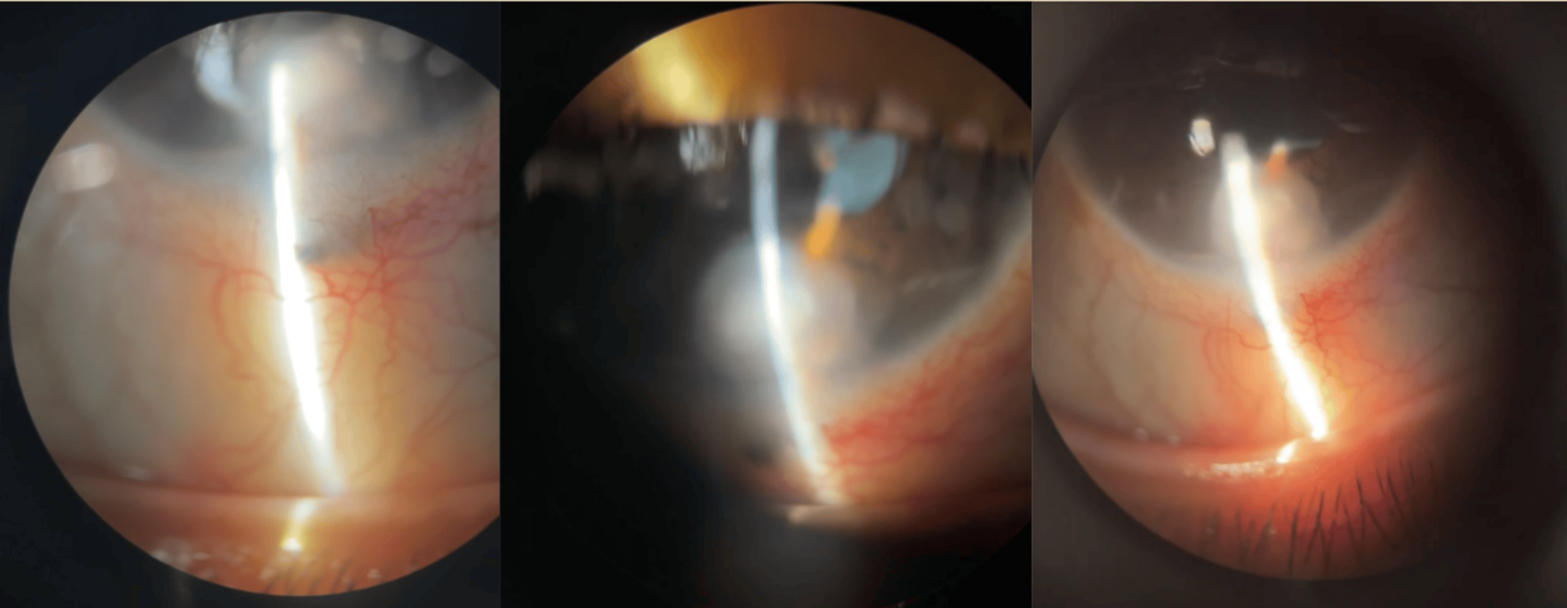
Slit lamp photos on day 32 showing significant improvement in scleritis and decreased infiltrate size with approximately 50% corneal thinning.

**Figure 10:**
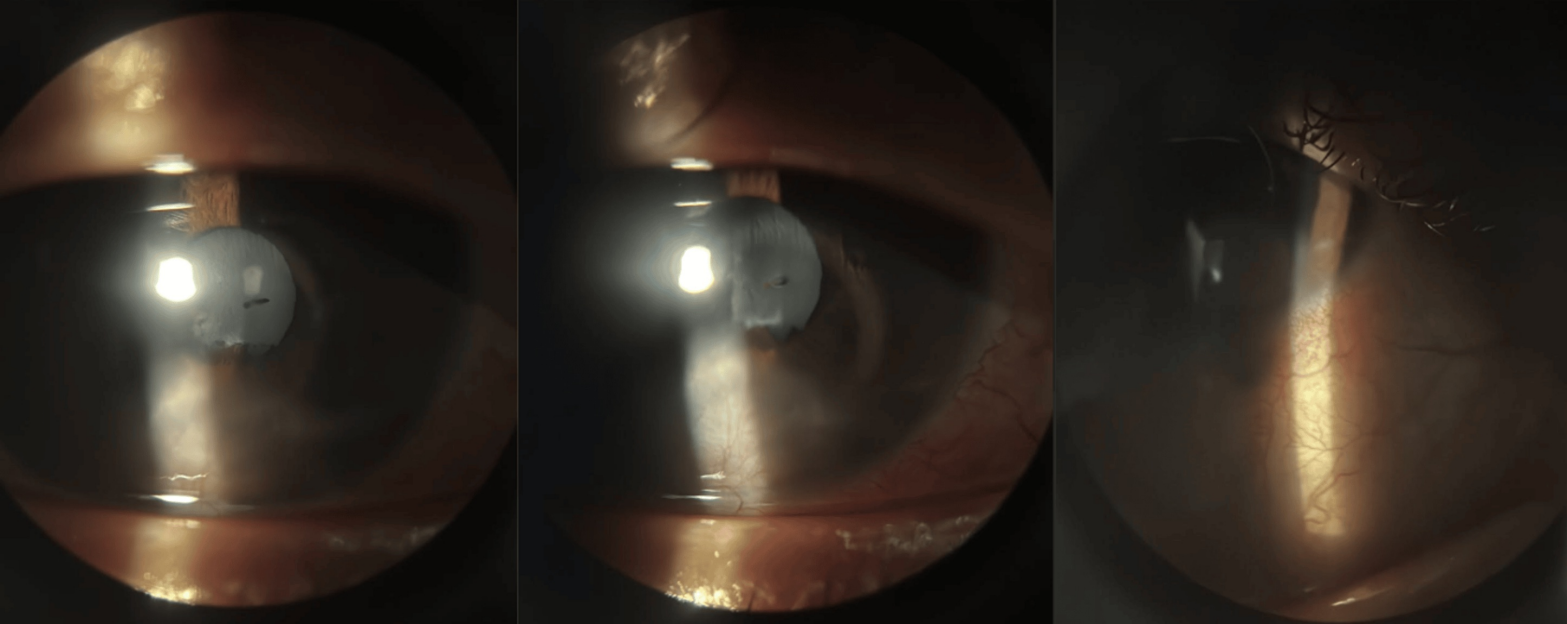
Slit lamp photos 3.5 months post-presentation showing area of corneal scarring and thinned sclera.

Course/outcome: By day 10, VA improved to 20/200 with reduced AC inflammation. At one month, VA was 20/40, pain resolved, and the cornea showed residual stromal thinning with peripheral neovascularization (Figure 9). At 3.5 months, VA was 20/30, IOP 16 OD on only dorzolamide/timolol, with mild residual scleral thinning and focal corneal scarring at the tap site (Figure 10) (Table 2).

**Table 2:** Timeline of clinical course.

Timepoint	Vision (OD)	IOP (mmHg)	Key Findings	Interventions
~1 wk post-I&D	—	—	Eye pain begins after MRSA abscess	—
2.5 mo post-I&D	20/50 → 20/150 (next day)	41	AC mass, corneal edema	Topical moxifloxacin, cyclopentolate; then prednisolone q2h; glaucoma drops
+3 days	LP	29	AC fibrin + hypopyon; nodular scleral thickening	Oral prednisone 60 mg
Retina/uveitis	LP	—	—	AC tap #1; prednisone ↑ to 80 mg; PO levofloxacin; indomethacin
Day 8 (cornea)	CF	36→21	Disrupted AC mass, hypopyon, corneal defect	AC tap #2; steroid taper
Day 9	—	—	—	Intravitreal vanc + ceftazidime
Day 10	20/200	20	Reduced AC inflammation	Continue levofloxacin + drops
1 month	20/40	—	Residual stromal thinning, PNV	—
3.5 months	20/30	16	Small scleral thinning, corneal scar	Dorzolamide-timolol BID OD

## Discussion

Anterior-predominant EE with scleral involvement. EE usually affects the posterior segment [1,2]. An AC abscess as the dominant finding is rare, and coexistent necrotizing scleritis is even less common. Infectious scleritis accounts for ~5-10% of scleritis cases and carries a high risk of perforation [3,4].

Source and laterality

Hematogenous seeding from skin/soft-tissue infection is a recognized pathway for EE [1]. The patient’s ocular involvement following a MRSA thigh abscess and thrombophlebitis exemplifies this mechanism. Right-eye involvement is consistent with the reported predilection due to direct carotid flow [2].

Diagnostic lesson

AC taps have lower sensitivity than vitreous samples, and false negatives are well documented [5,6]. Our case demonstrates the importance of repeat sampling when clinical suspicion is high.

Therapeutic lesson

Corticosteroids worsened inflammation until infection was proven. Once MRSA was identified, intravitreal vancomycin/ceftazidime and steroid taper led to recovery to 20/30. Recent MRSA EE reports confirm an aggressive course but potential for salvage if intravitreal coverage is prompt [7,8].

## Conclusions

Endogenous MRSA endophthalmitis presenting as an anterior chamber abscess with necrotizing scleritis is rare and easily mistaken for autoimmune disease. Early dismissal of an infectious etiology will lead to preventable vision loss. When clinical suspicion remains high, repeat diagnostic sampling should be performed despite negative initial results, and early targeted intravitreal therapy with steroid tapering can preserve vision.
